# Frequency controlled energy absorption in parametric mixing

**DOI:** 10.1038/s41598-026-39994-3

**Published:** 2026-02-18

**Authors:** Sean C. Chen, Lap K. Yeung, Keith Runge, Pierre A. Deymier, Yuanxun Ethan Wang

**Affiliations:** 1 Department of Electrical and Computer Engineering, University of California, Los Angeles, CA USA; 2 New Frontiers of Sound Science and Technology Center, The University of Arizona, Tucson, AZ USA

**Keywords:** Engineering, Materials science, Physics

## Abstract

This work presents the theory and experimental demonstrations of frequency-controlled energy absorption in parametric mixing circuits which may find applications in the design of tunable notch filters and frequency-selective surfaces. A time-varying capacitance model is used to describe the energy exchange among the pump, signal, and idler frequencies in parametric mixing circuits. Analytical derivations reveal that under specific frequency ordering, the system exhibits a positive conductance at the signal frequency that is correlated with the pump frequency, leading to measurable energy absorption. A fabricated on-chip circuit operating between 1.3 and 2.3 GHz serves as a physical representation of the theoretical model, experimentally validating the predicted energy transfer behavior. The results establish a unified framework linking circuit-level phenomena to the fundamental physics of energy redistribution in parametric systems.

## Introduction

Parametric mixing has been studied for more than half a century, with early works establishing its role in amplification, modulation, and frequency conversion^1–4^. In its simplest form, a reactive circuit element - typically a capacitance or inductance - is periodically modulated by a strong pump signal, introducing a time-varying reactance^5^. When an input signal interacts with this modulation, energy is exchanged among frequency components, generating a third frequency at the sum or difference known as the idler. This process enables a fundamentally lossless exchange of energy between the signal, idler, and pump, distinct from the dissipative mechanisms found in resistive-based systems. Recent research has extended parametric concepts to many different disciplines, including optical, superconducting, quantum, and mechanical systems^6–10^. In the optical domain, time-varying media and nonlinear interactions have been harnessed for processes such as wave mixing, frequency conversion, and optical parametric amplification. In these systems, absorption is typically regarded as detrimental - arising from intrinsic material loss rather than from the parametric process itself. In contrast, the present work explores absorption as an intrinsic outcome of energy redistribution in a time-varying system. Parallel developments in the quantum and RF domains have also deepened understanding of parametric energy exchange. The Josephson parametric amplifier (JPA) and related travelling-wave parametric amplifier (TWPA) architectures^11–18^ have demonstrated broadband operation through dispersion and impedance engineering, enabling enhanced dynamic range and quantum-limited noise performance. These efforts have extended the same underlying physics to a regime where signal and idler modes are strongly coupled across wide bandwidths.

In the RF and microwave regions, parametric processes have been applied in on-chip implementations, enabling compact and tunable devices for non-reciprocal signal flow, frequency translation, and filtering^19–23^. Most of these works focus on the amplification regime, in which a negative resistance is generated at the signal frequency, resulting in power gain. This gain can appear either as direct amplification or as conversion gain, where the mixed idler signal acquires higher power than the input. The physical mechanism is identical in both cases and depends on the idler satisfying the mixing condition $\:{\omega\:}_{i}={\omega\:}_{p}-{\omega\:}_{s}$. However, the complementary regime, where the idler frequency follows $\:{\omega\:}_{i}={\omega\:}_{p}+{\omega\:}_{s}$, has been far less explored. In this case, the system exhibits a positive resistance at the signal frequency, leading to energy absorption instead of gain. This phenomenon is part of the non-degenerate parametric amplification (NDPA) framework, where the direction of energy flow between the interacting frequencies determines whether the system amplifies or absorbs. Beyond its physical interest, this regime has practical implications for notch filtering and other frequency-selective energy control mechanisms.

In this work, we present a theoretical and experimental investigation of the absorption regime in a parametric mixing network. Specifically, this work has three main objectives: (i) to develop a circuit-based model to describe the exchange of energy among the signal, pump, and idler, showing that the observed positive conductance is intrinsically linked to the idler resonator’s quality factor and modulation strength, and can be interpreted a time-varying energy redistribution rather than conventional dissipative loss; (ii) to validate the theoretical framework through the implementation of a monolithic microwave integrated circuit (MMIC) operating between 1.3 and 2.3 GHz, designed as a physical representation of the theoretical model using varactor-based capacitance modulation to realize the predicted parametric interaction; and (iii) to experimentally demonstrate pump-controlled signal attenuation at the expected signal frequencies. Under appropriate pumping conditions, the chip exhibits measurable attenuation at the expected signal frequencies. Although the observed attenuation ($\:\sim3.5\:dB$) is modest due to the limited on-chip $\:Q$-factor, the result confirms the existence of the absorption effect. The findings establish a circuit-level foundation for energy exchange and selective attenuation in time-varying parametric systems.

The paper is organized as follows. The “Theory” section develops the analytical framework describing the underlying physics and its connection to parametric notch filtering. The “Model and Simulation” section presents simulation studies that model the absorption behavior and validate the theoretical relations. The “Chip Design and Experimental Verification” section details the MMIC implementation and demonstrates the observed energy absorption in the 1.3 to 2.3 GHz band. A “Discussion” section then contextualizes the experimental results, compares the proposed approach with existing notch-filtering techniques, and outlines practical pathways for improving attenuation depth. Finally, the “Conclusion” section summarizes the findings and discusses their broader implications for energy-preserving and time-modulated systems.

### Theory

Traditional parametric amplifiers leverage nonlinear elements with tunable reactance, such as varactor diodes, driven by a strong pump signal. When the pump frequency ($\:{\omega\:}_{p}$) is higher than both the idler ($\:{\omega\:}_{i}$) and signal ($\:{\omega\:}_{s}$) frequencies, parametric mixing produces negative resistance at the signal and idler frequencies. By terminating the unwanted difference sideband, single sideband mixing is achieved. This has been extensively studied and is well documented in prior work^19, 21^. The relevant frequency relation in this case is:

1 $$\:\begin{array}{c}{\omega\:}_{p}={\omega\:}_{i}+{\omega\:}_{s}\end{array}$$

Assuming all other mixing terms are terminated, the derived current–voltage matrix describing the parametric mixing interaction relates the signal and idler currents to their voltages as follows:

2 $$\:\begin{array}{c}\left(\begin{array}{c}{I}_{s}\\\:{I}_{i}^{*}\end{array}\right)=\left(\begin{array}{cc}j{\omega\:}_{s}{C}_{0}\:&\:j{\omega\:}_{s}{C}_{0}\gamma\:\\\:-j{\omega\:}_{i}{C}_{0}\gamma\:&\:-j{\omega\:}_{i}{C}_{0}\end{array}\right)\left(\begin{array}{c}{V}_{s}\\\:{V}_{i}^{*}\end{array}\right)\end{array}$$

From this matrix, the input impedance of the varactor at the signal frequency when there is resonance at the idler frequency is given by:

3 $$\:\begin{array}{c}{Y}_{s}=j{\omega\:}_{s}{C}_{0}-{\omega\:}_{s}{C}_{0}{Q}_{i}{\gamma\:}^{2}\end{array}$$

Where $\:{C}_{0}$ is the mean capacitance of the varactor, $\:\gamma\:$ is the capacitance modulation index, and $\:{Q}_{i}$ is the unloaded quality factor of the idler resonance. The presence of negative resistance at the signal frequency is given by the negative real value in Eq. (3).

In contrast, this paper focuses on the complementary case where the pump frequency and signal frequencies are lower than the idler frequency. Here, the mixing condition follows:

4 $$\:\begin{array}{c}{\omega\:}_{i}={\omega\:}_{s}+{\omega\:}_{p}\end{array}$$

For this case, the derived current-voltage matrix describing the parametric mixing interaction relates the signal and idler currents to their voltages as follows:

5 $$\:\begin{array}{c}\left(\begin{array}{c}{I}_{s}\\\:{I}_{i}\end{array}\right)=\left(\begin{array}{cc}j{\omega\:}_{s}{C}_{0}\:&\:j{\omega\:}_{s}{C}_{0}\gamma\:\\\:j{\omega\:}_{i}{C}_{0}\gamma\:&\:j{\omega\:}_{i}{C}_{0}\end{array}\right)\left(\begin{array}{c}{V}_{s}\\\:{V}_{i}\end{array}\right)\end{array}$$

The mixing action will be shown to create a positive conductance rather than negative at the signal frequency, $\:{\omega\:}_{s}$. The admittance seen at the signal frequency, $\:{Y}_{s}$, corresponding to the relationship given in Eq. (4) at idler resonance can be solved to be:

6 $$\:\begin{array}{c}{Y}_{s}=j{\omega\:}_{s}{C}_{0}+\frac{{\omega\:}_{s}{\omega\:}_{i}{C}_{0}^{2}{\gamma\:}^{2}}{{Y}_{i}+j{\omega\:}_{i}{C}_{0}}\end{array}$$

When a resonance is created at the idler frequency (i.e., adding a shunt inductor), this reduces to:

7 $$\:\begin{array}{c}{Y}_{s}=j{\omega\:}_{s}{C}_{0}+{\omega\:}_{s}{C}_{0}{Q}_{i}{\gamma\:}^{2}\end{array}$$

In this configuration, parametric mixing produces a positive conductance at the signal frequency $\:{\omega\:}_{s}$ given by the real value in Eq. (6). This added conductance models energy removal from the signal band through parametric mixing. When the conductance becomes sufficiently large, it also introduces an impedance mismatch that leads to increased reflection, together producing a stopband notch. The key to tunability lies in fixing the idler frequency with a resonator while sweeping the pump frequency. As the pump varies, the signal frequency that satisfies the mixing condition in Eq. (4) changes accordingly. Because the positive conductance is frequency-dependent and strongest at this condition, the signal experiences maximum attenuation at a tunable frequency set by the pump. As can be observed from Eq. (7), the equivalent input admittance of the modulated varactor diode is the parallel combination of the mean capacitor susceptance ($\:j{\omega\:}_{s}{C}_{0}$) and a positive real conductance ($\:{\omega\:}_{s}{C}_{0}{Q}_{i}{\gamma\:}^{2}$). Notably, the expressions for the negative and positive conductances derived in equations Eqs. (3) and  (7) are equal in magnitude but opposite in sign.

**Fig. 1:**
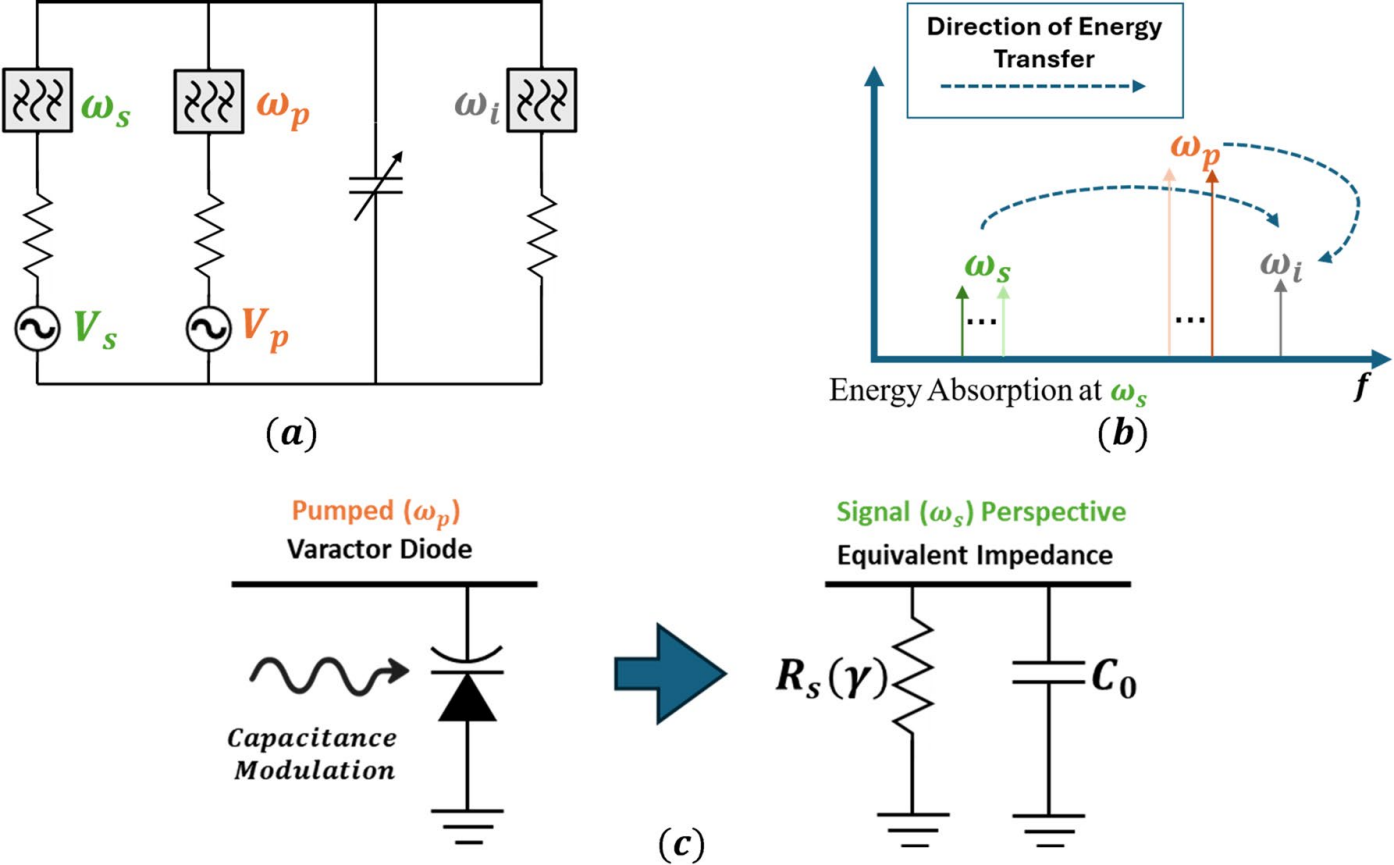
Illustrations of energy absorption in parametric mixing system. **a** Ideal circuit assuming perfect separation of signal ($\:{\omega\:}_{s}$), idler ($\:{\omega\:}_{i}$), and pump ($\:{\omega\:}_{p}$) performed with ideal bandpass filters for energy derivations. **b** Energy-distribution relationship, where dotted-arrows indicate direction of energy flow, indicating positive resistance at $\:{\omega\:}_{s}$. **c** Equivalent circuit model of impedance at the signal frequency assuming modulated capacitance in this configuration.

This added conductance can be conceptually represented as a resistive component in parallel with the varactor’s reactive admittance, as shown schematically in Fig. 1. The equivalent resistance, $\:{R}_{s}$, that models this absorption can be expressed as:

8 $$\:\begin{array}{c}{R}_{s}=\frac{1}{{\omega\:}_{s}{C}_{o}{Q}_{i}{\gamma\:}^{2}}\end{array}$$

It is important to note that the effective resistance $\:{R}_{s}$ appears as a shunt element in the idler admittance. Consequently, a decrease in$\:\:{R}_{s}$ corresponds to an increase in loss through increased conductance. The magnitude of this resistance is inversely proportional to both the capacitance modulation index, $\:\gamma\:$, and the quality factor of the resonator at the idler frequency, $\:{Q}_{i}$. Physically, a larger modulation index corresponds to stronger coupling between the signal and pump, enhancing the rate of energy exchange and increasing the equivalent conductance seen at the signal input. Similarly, a higher $\:{Q}_{i}$ implies more efficient energy storage in the idler resonance, further increasing the effective absorption seen at the signal port. The interaction therefore can be seen as an energy-dependent resistance, the value of which is controlled by both the strength of the pump modulation (i.e., pump signal), and the resonator quality at the idler frequency. In the limit of low $\:\gamma\:$ or low $\:{Q}_{i}$, this shunt resistance becomes large, and the circuit approaches a purely reactive behavior with minimal energy transfer. Conversely, as $\:\gamma\:$ and $\:{Q}_{i}$ increase, the impedance becomes dominated by its real component, producing a measurable attenuation or “notch” in the input signal.

In an ideal lossless system, the energy exchange among the signal, pump, and idler obeys the Manley-Rowe relations^4^, which dictate the conversion limits of the parametric process. For three-wave mixing in the mixing condition described by Eq. (4), the relations impose the following criteria:

9 $$\:\begin{array}{c}\frac{{P}_{i}}{{\omega\:}_{i}}=-\frac{{P}_{p}}{{\omega\:}_{p}}=-\frac{{P}_{s}}{{\omega\:}_{s}},\end{array}$$

where $\:{P}_{k}$ denotes the average power flowing out of the nonlinear element at frequency, $\:{\omega\:}_{k}$. When parametric mixing is enabled, energy is coupled out of the signal band in accordance with these relations. From the signal-frequency perspective, this manifests as an effective shunt resistance in the equivalent circuit, representing net energy removal at the signal frequency. As the idler-frequency power ($\:{P}_{i}$) flowing out increases, the signal-frequency power flowing in $\:({P}_{s}$) must increase proportionally (by the factor $\:{\omega\:}_{s}/{\omega\:}_{i}$), consistent with Manley-Rowe energy exchange. Here, an increase in the magnitude of negative $\:{P}_{s}$ corresponds to increased energy removal from the signal port.

### Model and simulation

For parametric notch-filtering to be effective, the pump, idler, and signal paths must remain well isolated. To achieve this, simulation model splits the single-ended input RF signal ($\:{\omega\:}_{s}$) into two paths. Each path mixes with the first pump ($\:{\omega\:}_{p}$) which is applied in relative antiphase between the two branches. This arrangement generates a differential idler component ($\:{\omega\:}_{i}$) that is confined to the resonant tank formed by the inductor and the varactor capacitance. The symmetry of the circuit establishes virtual grounds at the RF combination nodes, thereby isolating the idler from the signal path and minimizing undesired feedthrough among the three frequencies.

**Fig. 2:**
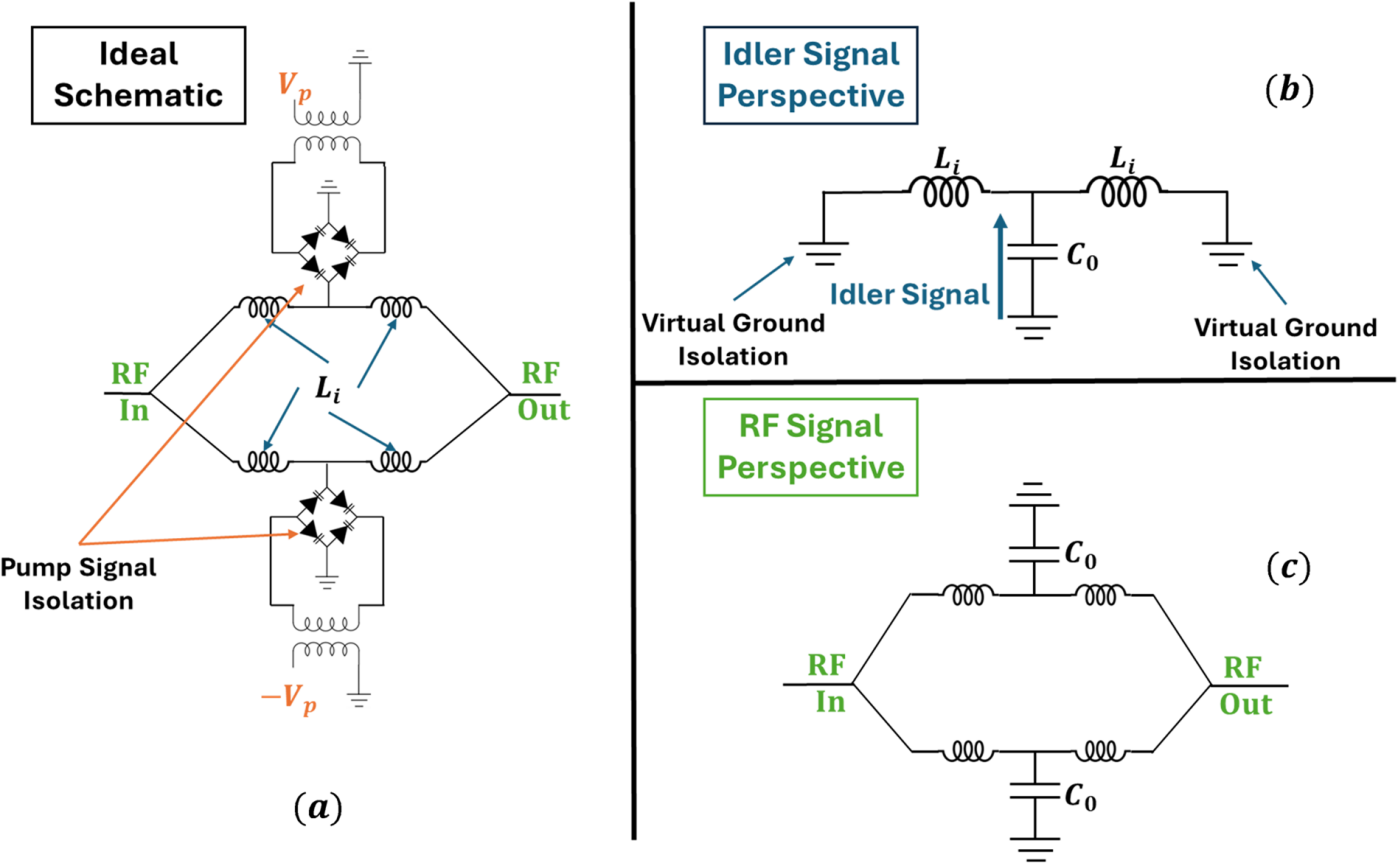
Ideal circuit mockup for simulations. **a** Ideal schematic. **b** Idler signal perspective, showcasing LC resonant tank and isolation from input/output terminals due to vertical grounds. **c** RF signal perspective, showcasing LC lowpass filter behavior.

From the signal’s perspective, the circuit behavior transitions depending on the pump state. When the pumps are inactive, the circuit appears as a simple inductor line shunted with capacitors - effectively behaving as a lowpass filter (Fig. 2c). When the pumps are on, parametric mixing introduces a time-varying susceptance that gives rise to a positive conductance term at the signal frequency, as described by Eq. (7). This added conductance absorbs a portion of the incident RF power and produces a tunable attenuation notch in the transmission response. The diode-bridge configuration further isolates the pump from the RF port, and vice versa, ensuring that the observed notch originates purely from the parametric interaction rather than direct loading.

To validate this mechanism, both the theoretical attenuation estimates and circuit-level simulations were carried out using an equivalent small-signal lumped-element model. The idler resonance frequency $\:{\omega\:}_{i}$ was fixed at 4 GHz, and the resonant network was implemented entirely using lumped elements. The inductor value was chosen to resonate with the mean varactor capacitance $\:{C}_{0}=0.71\mathrm{\:pF}$ according to $\:{f}_{i}=1/\left(2\pi\:\sqrt{LC}\right)$, yielding an inductance of $\:{L}_{i}=2.28\mathrm{\:nH}$. The unloaded quality factor of the LC idler network was set to $\:{Q}_{i}\approx\:100$ unless otherwise stated. Varactor diodes were modeled using GCS D10 MMIC process parameters.

**Fig. 3:**
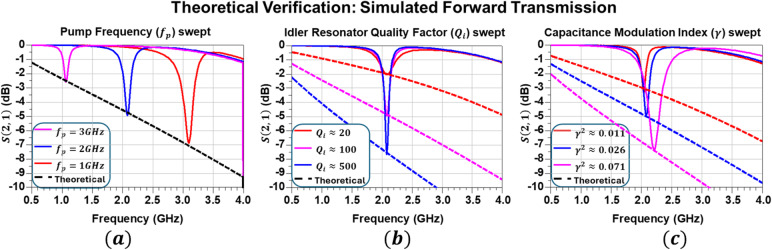
Simulated transmission coefficient $\:S\left(\mathrm{2,1}\right)$ of the ideal circuit mockup. **a** Tunable attenuation notch for pump frequencies ($\:{f}_{p}=\left\{\mathrm{1,2},3\right\}\:GHz$). **b** Dependence of attenuation depth on the idler-resonator quality factor ($\:{Q}_{i}$). **c** Dependence of attenuation depth on the capacitance modulation index, $\:\gamma\:$ (plotted as $\:{\gamma\:}^{2}$). Dashed curves indicate attenuation predicted by the analytical model.

The simulated transmission responses $\:S\left(\mathrm{2,1}\right)$ are shown in Fig. 3 for three complementary parameter sweeps. In Fig. 3a, the pump frequency was swept over $\:{f}_{p}=\{\mathrm{1,2},3\}$ GHz with a fixed pump power of 15 dBm per port, corresponding to a capacitance modulation index of approximately $\:\gamma\:=0.16$. According to the mixing condition $\:{\omega\:}_{i}={\omega\:}_{s}+{\omega\:}_{p}$, this sweep produces a tunable absorption band spanning 3 GHz down to 1 GHz in the signal frequency $\:{f}_{s}$, resulting in a moving attenuation notch. In Fig. 3b, the pump frequency was fixed at $\:{f}_{p}=3\mathrm{\:GHz}$, while the idler-resonator quality factor was swept over $\:{Q}_{i}\approx\:\left\{\mathrm{20,100,500}\right\}$, with the pump power held constant at 15 dBm ($\:\gamma\:\approx\:0.16$). The results show a monotonic increase in attenuation depth with increasing $\:{Q}_{i}$, consistent with the analytical dependence predicted by Eq. (7).

Finally, in Fig. 3c, the capacitance modulation index was varied by sweeping the pump power over $\:{P}_{p}=\left\{\mathrm{11,15,19}\right\}$ dBm, corresponding to $\:\gamma\:\approx\:\left\{\mathrm{0.10,0.16,0.27}\right\}$, with $\:{\gamma\:}^{2}$ indicated in the legend. Increasing the pump power enhances the modulation strength and deepens the attenuation notch, while also slightly shifting the mean capacitance $\:{C}_{0}$ and consequently the center frequency of the stopband. In all cases, the predicted insertion loss at resonance agrees closely with the simulated attenuation, confirming that the apparent resistive behavior originates from the positive-conductance term $\:{\omega\:}_{s}{C}_{0}{Q}_{i}{\gamma\:}^{2}$.

### Chip design and experimental verification

**Fig. 4:**
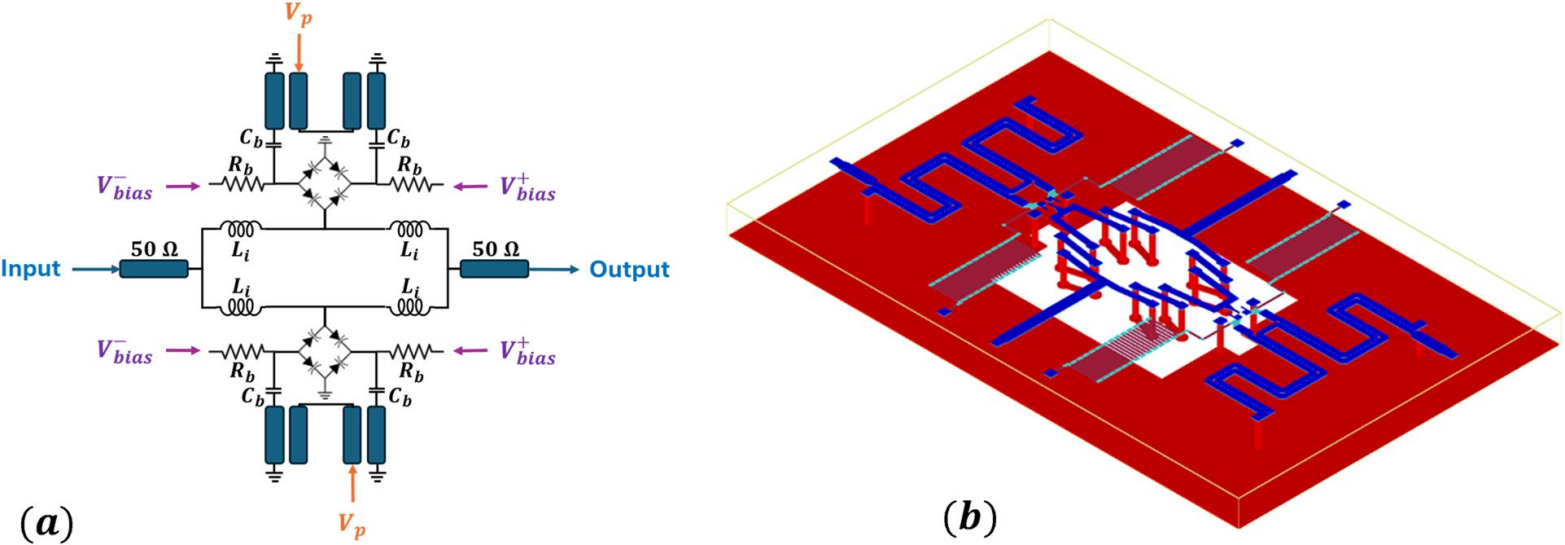
MMIC layout design. **a** Circuit schematic. **b** Chip layout employing Marchand balun structures to differentially inject the pump signal.

**Fig. 5:**
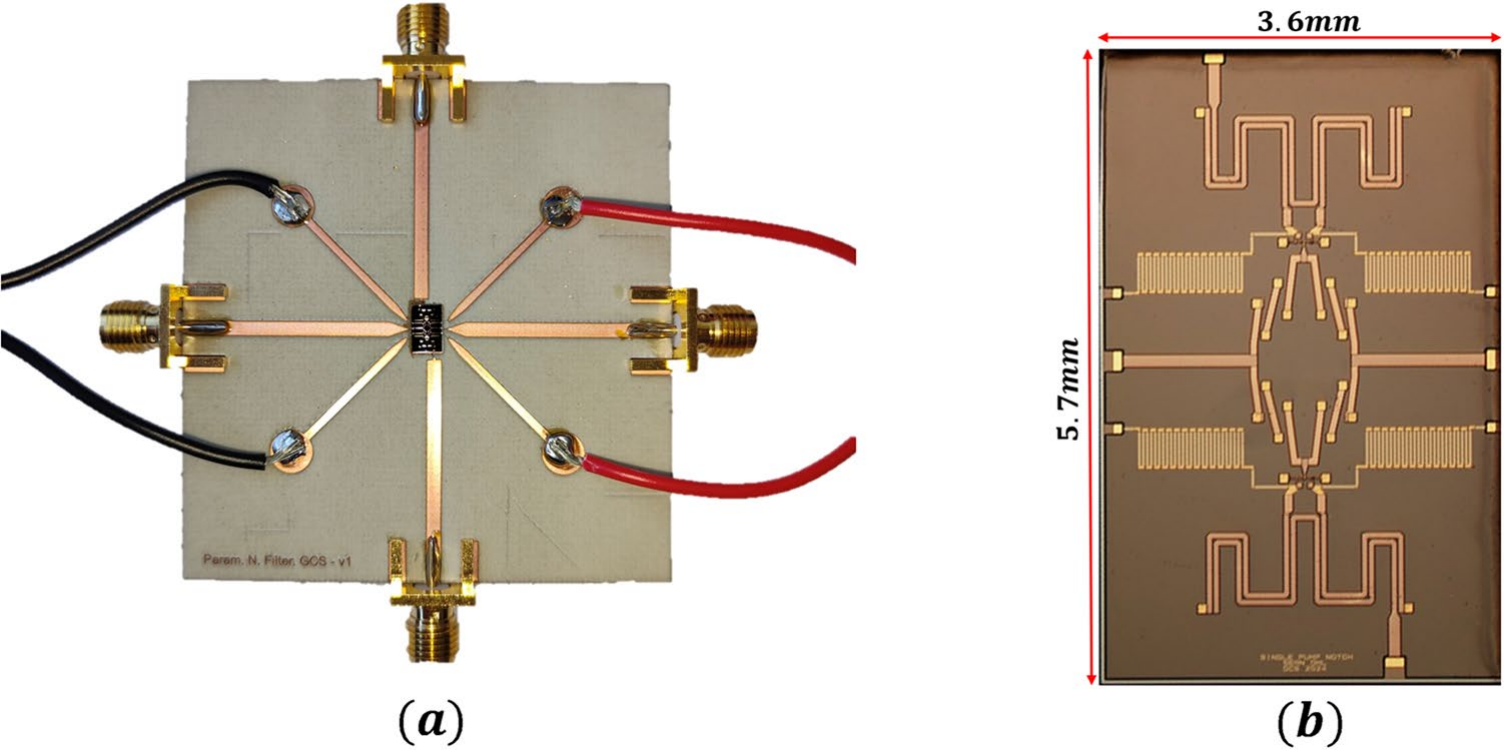
Measurement hardware and fabricated device. **a** PCB test fixture used for RF characterization. **b** Optical micrograph of the fabricated chip.

The monolithic microwave integrated circuit (MMIC) designed to verify the theoretical model is shown schematically in Fig. 4a, with the corresponding on-chip layout in Fig. 4b. The circuit implements the lumped resonant topology analyzed in the previous section, with additional components optimized for on-chip measurement and pump injection. A Marchand balun is integrated at the input of the pump line to provide differential excitation of the two varactor branches while maintaining broadband coupling. The bias resistors function as RF blocks, isolating the DC bias network from the high-frequency signal paths. The input and output transmission lines are designed for 50$\:{\Omega\:}$ characteristic impedance to support broadband propagation and enable direct connection to external measurement equipment. The fabricated MMIC is mounted and wire-bonded onto a 32-mil Rogers 4003 C printed circuit board with matched 50$\:{\Omega\:}$ microstrip traces leading to the RF ports. The assembled board is shown in Fig. 5a, while an optical micrograph of the mounted chip is shown in Fig. 5b. A continuous-wave pump signal was injected differentially through the on-chip balun, while the input RF signal is applied through a standard SMA connector.

**Fig. 6:**
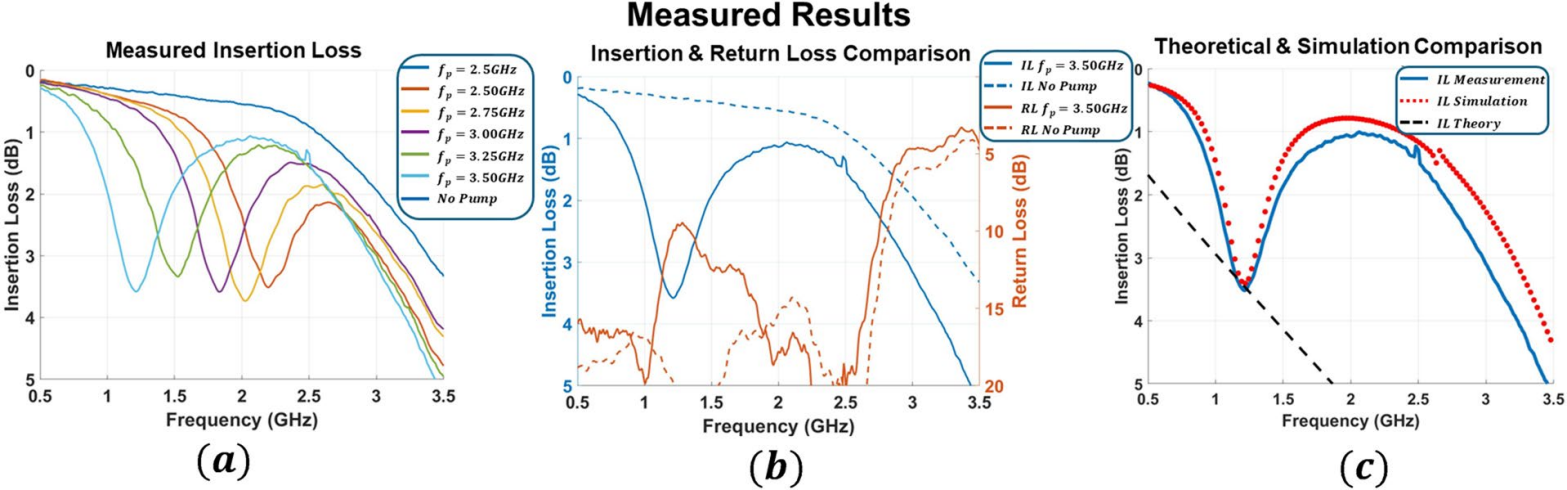
Measured RF performance **a** Measured insertion loss as the pump frequency$\:\:{f}_{p}$ is swept from 2.5 to 3.5 GHz. **b** Measured insertion and return loss at $\:{f}_{p}=3.5$ GHz, compared with the case without pump injection. **c** Measured insertion loss at $\:{f}_{p}=3.5$ GHz compared with simulation and theory.

The measured transmission characteristics are shown in Fig. 6a, where the pump frequency is swept from 2.5 GHz to 3.5 GHz. As predicted by the theoretical and simulated models, the response exhibits a tunable attenuation notch corresponding to the energy-absorption regime. The measured attenuation depth averages 3.5 dB, with a relatively constant bandwidth of 220 MHz, and the center frequency tracking the pump sweep in agreement with the mixing relation $\:{\omega\:}_{i}={\omega\:}_{s}+{\omega\:}_{p}$. To further verify the absorption mechanism, Fig. 6b presents the measured insertion and return loss for a fixed pump frequency while sweeping the input signal. The return-loss parameter ($\:{S}_{11}$) shows partial reflection of the absorbed energy back toward the source. The relative distribution of transmitted, reflected, and absorbed energy can be estimated directly from the measured scattering parameters in Fig. 6b. At the fixed pump frequency of 3.5 GHz, the measured insertion loss at the notch frequency is approximately 3.6 dB, corresponding to 66.1% of the incident signal power being removed from transmission. At the same frequency, the measured return loss is approximately 10.1 dB, indicating that about 9.8% of the removed power is reflected. The remaining portion of the lost power is absorbed through the parametrically generated conductance, with a fraction dissipated through finite substrate, metal, and device losses. Figure 6c further compares the measured insertion loss at $\:{f}_{p}=3.5$ GHz with circuit-level simulation and the theoretical prediction obtained from the equivalent parametrically generated conductance in Eq. (7). The theoretical curve is computed by converting the analytically derived positive conductance, based on simulated $\:{f}_{p}$, $\:{Q}_{i}$, and $\:\gamma\:$ values, into an equivalent insertion loss at the signal frequency. Good agreement is observed among all three results, with a depth deviation of approximately 2.6% between measurement and simulation and approximately 2% between measurement and theory at the notch frequency.

Overall, the measurements validate the analytical and simulated models: as the pump frequency is varied, the absorption notch shifts accordingly, demonstrating a direct experimental demonstration of parametric energy exchange in a time-varying circuit. The measured transmission and reflection behavior confirms the presence of a positive conductance at the signal frequency, consistent with the theoretical prediction of a pump-controlled resistive component $\:{R}_{\mathrm{s}}=1/\left({\omega\:}_{s}{C}_{0}{Q}_{i}{\gamma\:}^{2}\right)$. Despite the modest attenuation depth - limited by the achievable on-chip $\:{Q}_{i}$ - the observed behavior confirms that energy absorption can be realized through purely reactive parametric modulation.

## Discussion

The primary goal of this work is to showcase parametric absorption as a mechanism for frequency-controlled energy absorption using reactive mixers, and to demonstrate this effect experimentally in an RF circuit. An immediate and natural application of this phenomenon is in notch-filtering technology. As summarized in Table 1, the proposed approach occupies a distinct position relative to conventional notch-filtering techniques. Whereas passive, varactor-tuned, switched, and absorptive notch filters rely on resonant mismatch or explicit resistive dissipation to achieve frequency-selective attenuation, the present work leverages pump-controlled parametric energy absorption enabled by time-varying reactance. The prototype presented here is not intended to represent a performance-optimized filter implementation, but rather to experimentally demonstrate a complementary and previously unexplored mechanism for frequency-controlled energy removal in RF circuits.

The achievable notch depth in the present implementation is limited by the effective idler quality factor$\:\:{Q}_{i}$, which is constrained by finite on-chip losses associated with the resonator, metal routing, and substrate dissipation. Importantly, this limitation is practical rather than fundamental, and does not detract from the validity of the parametric absorption framework itself. One practical path toward improving notch depth is to enhance the effective idler quality factor through loss compensation using negative resistance. This can be achieved by introducing an active element - such as an oscillator or another parametric device - that provides controlled negative conductance at the idler frequency. By partially or fully canceling intrinsic resonator losses, the effective$\:\:{Q}_{i}$ can be increased, directly enhancing the depth and sharpness of the attenuation notch. Such Q-enhancement techniques have been widely explored in parametric and active resonator systems and are well aligned with the operating principles of the proposed architecture.^21,24^.

Alternatively, for applications requiring a fully passive and compact solution, integration with acoustic-wave resonator technology offers a compelling approach. Thin-film bulk acoustic resonators (FBARs) provide exceptionally high quality factors at GHz frequencies within a small footprint, making them well suited to serve as the idler resonance in a parametric absorption network. Prior work has demonstrated successful integration of microwave circuits with acoustic-wave devices, as well as hybrid electrical-acoustic filtering topologies. Incorporating such high-Q resonators would directly improve $\:{Q}_{i}$ without introducing active components, enabling substantially deeper notches while preserving the inherent tunability of the parametric scheme^25,26^.

**Table 1:** Comparison of representative notch-filtering and Attenuation approaches.

Approach	Attenuation mechanism	Tunability method	Bandwidth control	Implementation	3dB fractional bandwidth	Area mm^2^
Passive notch filter	Resonant mismatch	None	Fixed by resonator Q	Lumped / distributed resonators	$\:1\sim5\%$ ^27a^	–
Varactor-tuned notch filter	Resonant mismatch	Electrical bias	Limited by varactor tuning	Varactors + resonators	$\:10\sim16\%$ ^28b^	PCB Scale
Absorptive notch filter	Resistive absorption	Electrical bias	Set by lossy resonator	Resistor-loaded networks	$\:3\sim6\%\:$ ^29^	44.7 × 11.85
Switched notch filter	Resonant mismatch	MEMs switching	Discrete	Switched resonators	$\:42\sim52\%$ ^30^	PCB Scale
		PIN diode switching		EBG-based structure	$\:8\sim9\%$ ^31b^	PCB Scale
This work	**Parametric energy ** **absorption**	**Pump frequency**	**Set by idler** $\:{\boldsymbol{Q}}_{\boldsymbol{i}}$	**Parametric mixers**,**time-varying reactance**	$\:10\sim18\boldsymbol{\%}$	**5.7 × 3.6**

^**a**^Conventional fixed resonator-based RF notch filters typically exhibit narrow fractional bandwidths determined by the resonator Q, often on the order of a few percent. They also cover diverse lumped and distributed implementations, for which a representative physical area is not well defined.^**b**^Calculated visually from plots because not explicitly reported. Significant values are in [bold].

## Conclusion

This work demonstrates both theoretically and experimentally the phenomenon of energy absorption in parametric mixing systems. Using a time-varying capacitance model, it is shown that when $\:{\omega\:}_{i}={\omega\:}_{s}+{\omega\:}_{p}$, a positive conductance appears at the signal frequency - the counterpart to the negative resistance commonly seen in parametric amplification. The derived admittance expression reveals that the absorption strength depends on the capacitance modulation index ($\:\gamma\:$) and the idler-resonator quality factor ($\:{Q}_{i}$). Circuit simulations confirm these dependencies, and an MMIC prototype operating from 1.3 to 2.3 GHz experimentally verifies the effect. The measured response exhibits a tunable attenuation notch of about 3.5 dB, consistent with the theoretical prediction of pump-controlled energy absorption. These results establish a direct link between parametric energy exchange and tunable notch filtering, providing a foundation for future reconfigurable and time-modulated circuit designs that exploit controlled signal-band attenuation without relying on conventional dissipative loss elements.

## Methods

*Simulations* Circuit-level simulations were carried out in Keysight ADS schematic to evaluate the equivalent impedance and energy absorption behavior based on the small-signal admittance formulation developed in Eq. (7). To generate the theoretical attenuation curve shown in Fig. 3, the analytically derived effective conductance was first computed using the closed-form expression and then incorporated into an idealized circuit model by shunting this conductance with the signal-frequency capacitance, consistent with the architecture shown in Fig. 2c. Foundry-provided PDK models were used for the varactor diodes in the non-linear circuit simulations, while passive components - including spiral inductors, DC blocking capacitors, transmission lines, and the Marchand balun - were designed and verified using full-wave electromagnetic (EM) simulations. Unless otherwise stated, circuit-level simulations employed lumped-element representations of the on-chip resonant network, with parameter sweeps applied to the pump frequency, idler-resonator quality factor, and capacitance modulation index as described in the main text.

*Design and fabrication* The monolithic microwave integrated circuit (MMIC) prototype was fabricated using a 1-µm InGaAs heterojunction bipolar transistor (HBT) process on a 150-µm GaAs substrate. Global Communication Semiconductor (GCS) D10 varactor models and diodes were employed as the nonlinear reactive elements to provide the time-varying capacitance modulation required for the parametric mixing process. On-chip spiral inductors, thin-film resistors, and metal-insulator-metal (MIM) capacitors were implemented using the top metal layers of the process. DC blocking capacitors were integrated between the Marchand balun and the varactor diode bridge to isolate the RF pump path from the DC bias network. Although the on-chip inductor and varactor capacitance values alone do not yield the target idler resonance according to $\:{f}_{i}=1/2\pi\:\sqrt{LC}$, the effective idler resonance is determined by the total loaded capacitance at the idler node, which includes a substantial parasitic contribution from the Marchand balun and associated routing structures. Key component values and geometries are summarized in Table 2.

**Table 2:** On-chip component values, geometries, and design parameters for the MMIC parametric absorber prototype.

Component	Parameter	Value	Unit	Implementation
Varactor diode	Mean capacitance ($\:{C}_{0}$)	0.26	pF	GCS D10 varactor
	Capacitance modulation index ($\:\gamma\:$)	0.44	–	Pump-controlled: $\:\sim$30dBm
	Bias voltage	− 3.5	V	DC reverse bias
Idler inductor	Inductance ($\:{L}_{i}$)	1.42	nH	On-chip vertical
	Q-factor @ $\:{f}_{i}$	25	–	
Idler resonator	Target resonance ($\:{f}_{i}$)	4.8	GHz	LC tank
	Unloaded resonator Q-factor ($\:{Q}_{i}$)	13	–	Lumped LC
Bias resistor	Resistance ($\:{R}_{b}$)	15	k$\:{\Omega\:}$	Thin-film resistor
DC block capacitor	Capacitance ($\:{C}_{b}$) @ $\:{f}_{i}$	5	pF	MIM capacitor
RF transmission lines	Characteristic impedance	50	$\:{\Omega\:}$	On-chip microstrip
Pump balun	Center passband frequency @ 50$\:{\Omega\:}$	3	GHz	On-chip Marchand balun
Die size	Chip area	3.6 mm x 5.7 mm	mm²	GaAs MMIC
Chip substrate	Thickness	150	µm	GaAs

*Measurements* The fabricated chip was mounted and wire-bonded onto a 32-mil Rogers 4003 C printed circuit board with 50 $\:{\Omega\:}$ microstrip traces leading to the RF input and output pads. The input and pump signals were delivered through standard SMA connectors, while − 3.5 V DC bias voltages were supplied through external cables and isolated using on-board bias networks. S-parameter characterization was conducted using a Keysight PNA-X N5241A vector network analyzer. A continuous-wave pump signal with power levels up to roughly $\:\sim$30 dBm was injected through an external amplifier and swept from 2.5 to 3.5 GHz. The resulting transmission ($\:{S}_{21}$) and reflection ($\:{S}_{11}$) parameters were measured across the 0.5$\:\sim$3.5 GHz signal band to observe the energy-absorption behavior.

For both the simulations and measurement, although the applied pump power levels are relatively high, most of the incident pump power is reflected due to the impedance mismatch between the 50 $\:{\Omega\:}$ pump source and the predominantly capacitive load presented by the varactor network. The selected pump power levels ensure sufficient capacitance modulation while maintaining the RF voltage swing entirely within the reverse-bias operating region of the varactors.

## Data Availability

The datasets generated and analyzed during the current study are available from the corresponding author upon reasonable request.
